# Range-based volatility, expected stock returns, and the low volatility anomaly

**DOI:** 10.1371/journal.pone.0188517

**Published:** 2017-11-30

**Authors:** Benjamin M. Blau, Ryan J. Whitby

**Affiliations:** Department of Economics and Finance, Jon M. Huntsman School of Business, Utah State University, Logan, Utah, United States of America; Universidad Veracruzana, MEXICO

## Abstract

One of the foundations of financial economics is the idea that rational investors will discount stocks with more risk (volatility), which will result in a positive relation between risk and future returns. However, the empirical evidence is mixed when determining how volatility is related to future returns. In this paper, we examine this relation using a range-based measure of volatility, which is shown to be theoretically, numerically, and empirically superior to other measures of volatility. In a variety of tests, we find that range-based volatility is negatively associated with expected stock returns. These results are robust to time-series multifactor models as well as cross-sectional tests. Our findings contribute to the debate about the direction of the relationship between risk and return and confirm the presence of the low volatility anomaly, or the anomalous finding that low volatility stocks outperform high volatility stocks. In other tests, we find that the lower returns associated with range-based volatility are driven by stocks with lottery-like characteristics.

## Introduction

Much of financial economics is grounded on the assumption that risk and return are positively related. Traditional asset pricing theory rests on the assumption that rational investors will have preferences for low levels of risk [1]–[3]. Less demand for riskier assets implies lower stock prices and higher future returns. Initial tests of the relationship between risk and return seem to confirm this fundamental idea. For instance, a positive time-series relationship between market volatility and value-weighted market returns has been shown when ARIMA and GARCH models are used to calculate volatility [4]. Similar results are found for aggregate idiosyncratic volatility [5], [6]. However, conflicting results regarding the cross-sectional association between volatility and future returns have been shown in other research. When examining volatility and current returns at the stock level, a negative relation between expected returns and volatility—especially idiosyncratic volatility, has been documented for both U.S. and international markets [7], [8]. Volatility is generally measured as the standard deviation of returns, or residual returns, where residuals are obtained from daily Fama and French regressions [9]. On the other hand, Fu [10] shows that volatility is time-varying and that, when forecasting volatility using exponential GARCH models, the lead-lag, cross-sectional relation between volatility and stock returns becomes positive. This more recent literature seems to indicate that the direction of the relation between risk and return at the firm level depends on how volatility is measured.

In this study, we contribute to the debate by examining a range-based measure of volatility, which has been shown to be theoretically, numerically, and empirically superior to other measures of volatility in its efficiency [11]. Compared to other measures of volatility, range-based volatility is also distributed more normally and is robust to microstructure issues that are often problematic in volatility estimation.

We conduct a series of tests to determine the relation between next-month returns and the natural log of the difference between the highest price and the lowest price during a particular month, which we denote as range-based volatility hereafter. After sorting stocks into five value-weighted portfolios constructed on range-based volatility, we find that next-month average returns are monotonically decreasing. The return difference between extreme portfolios is statistically and economically significant. For instance, the difference between extreme portfolios is approximately 1.1% per month. In additional tests, we estimate portfolio alphas from CAPM and other multifactor models [9], [12]. Results show that alphas are generally decreasing across increasing range-based volatility portfolios. We again find that differences in alphas between extreme portfolios are statistically significant and economically meaningful. For example, when using the Fama and French [9] three-factor model, the alpha in the lowest range-based volatility portfolio is 92 basis points per month. The high-minus-low difference yields an alpha of slightly more than 1% per month. Our portfolio analysis, therefore, documents a significant, negative return premium associated with range-based volatility.

In a series of other tests, we examine the cross-sectional relationship between range-based volatility and next-month returns. Using a number of different Fama-MacBeth [13] regressions, we find that, after controlling for beta, market cap, book-to-market ratios, momentum, and illiquidity [14], range-based volatility produces a negative estimate that is both statistically and economically significant. In economic terms, a one-standard deviation increase in range-based volatility is associated with a 35 basis point reduction in next-month returns. In our analysis, the momentum premium, or the return premium associated with a one-standard deviation increase in past returns from month t-12 to t-2, is 32 basis points per month. Thus, the negative return premium associated with range-based volatility is of the same magnitude as the positive momentum return premium. Interestingly, other tests do not reveal a significant return premium associated with our measure of idiosyncratic volatility. When controlling for idiosyncratic volatility, however, we still observe the negative return premium for range-based volatility. In fact, the magnitude of the return premium is unchanged whether or not we control for idiosyncratic volatility.

In our second set of tests, we begin to explore why we observe underperformance in stocks with the greatest price range. We first examine this cross-sectional, negative return premium for various subsamples based on other measures of risk, specifically, idiosyncratic volatility and beta. While we find that the negative return premium associated with range-based volatility holds in each of the idiosyncratic volatility subsamples, we find strong evidence that the premium is strongest in stocks with the highest idiosyncratic volatility. We also find some evidence that stocks with high betas seem to drive the negative range-based volatility premium. These results suggest that the negative association between range-based volatility and next-month returns is partially explained by higher levels of risk.

Next, we attempt to further identify the explanation for the peculiar negative return premium found in range-based volatility. A number of studies have attempted to provide explanations for this relationship between volatility and future returns. For instance, Baker and Wurgler [15] argue that since the performance of institutional investors is benchmarked against an index, these investors have disincentives to arbitrage low volatility stocks. A variety of other arguments that stem from the limits to arbitrage related to low volatility stocks have also been examined [16], [17]. Another potential explanation for the observed negative relationship between risk and return is based on the psychology literature. Prospect theory is an alternative to expected utility theory [18]. According to prospect theory, individuals tend to overweight the tails of return distributions and have a stronger aversion to losses than preferences for gains. The application of prospect theory to finance shows that investor preferences for stocks that resemble lotteries can theoretically lead to price premiums and subsequent underperformance of stocks [19]. Empirical research tends to support this prediction [20]–[24]. An alternative argument is that volatility reflects an important lottery-like property and results in the underperformance of stocks with higher volatility (and other lottery-like characteristics) [25]. More recently, there has been evidence that the underperformance of stocks with high levels of systematic risk is driven by investor demand for lottery stocks [26].

We test whether the return premium associated with range-based volatility is driven by stocks that are most likely to resemble lotteries. Using the various lottery characteristics from the prior literature, we find that the range-based volatility return premium is driven by stocks that most resemble lotteries. These results are robust to different lottery classifications [25], measures of expected idiosyncratic volatility [21], and the measure of maximum daily returns [22]. Combined, our results suggest that not only is there a negative return premium associated with range-based volatility, but the premium appears to be driven by stocks that have lottery-like characteristics.

By showing that range-based volatility has a negative effect on future returns, our study contributes to the debate about the fundamental relation between risk and return. To the extent that range-based volatility is, in fact, a superior measure of volatility [11], our findings indicate that generally speaking, investors overpay for risky assets. Although some research suggests that stocks with high volatility should have higher expected returns because investors cannot fully diversify away from the firm-specific risk in their portfolios [27], our findings support the growing body of evidence that documents a negative return premium in stocks with higher volatility. Further, the second part of our analysis provides additional support for the argument that preferences for more volatile stocks are directly associated with preferences for stocks that look like lotteries [25], [26].

## Data description

The data used throughout the analysis come from several sources. From the Center for Research on Security Prices (CRSP), we obtain stock prices, returns, trading volume, shares outstanding at the daily and monthly level. From Wharton Research Data Services (WRDS), we gather daily and monthly Fama-French factors [9], [12]. We also obtain the annual book value of equity for each stock. We note that to be included in our sample, we require the book value of equity to be positive. The sample time period extends from 1980 to 2012. After combining the data from each of these sources for our time period, we are left with more than 19,000 unique stocks and nearly 1.88 million stock-month observations.

Using the highest ask price and the lowest bid price during a particular month, we calculate the variable of interest, range-based volatility, as the natural log of the difference between the high price and the low price during a particular month (*Ln(Price Range)*). We estimate other measures of risk: idiosyncratic volatility (*IdioVolt*) and *Beta*. *IdioVolt* is calculated by first estimating the following equation using daily data for each stock in our sample.

$$R_{i,t}-\;R_{f,t}=\alpha +\beta_{MKT}MKT_{t}+\beta_{HML}HML_{t}+\beta_{SMB}SMB_{t}+\beta_{UMD}UMD_{t}+\varepsilon_{i,t}$$ (1)

The dependent variable is the daily excess return for each stock *i* (over the yield on one-month T-bills). The independent variable includes *MRP*, which is the market risk premium, or the excess return of the market less the risk-free rate. *SMB* is the small-minus-big return factor while *HML* is the high-minus-low return factor. *UMD* is the up-minus-down, momentum factor. Here, the subscript *t* represents a particular day in our sample time period. *IdioVolt* is the standard deviation of the daily residual returns *ε*_*i*,*t*_ in each month. *Beta* is obtained from estimating a variant of Eq (1), where we restrict *β*_*HML*_ *= β*_*SMB*_ *= β*_*UMD*_ = 0. We note that both *Beta* and *IdioVolt* are estimated using rolling six-month windows to allow for a sufficient number of observations to preserve accuracy. *Size* is stock *i*’s monthly market capitalization on the last day of each month. *B/M* is the book-to-market ratio using our monthly *Size* variable and the annual book value of equity from Compustat. *Momentum* is the cumulative return from month *t-12* to *t-2* for each stock *i*. *Illiquidity* is calculated as the average daily ratio of the absolute value of the return to trading volume (in 100,000s) [14]. Multiple papers have discussed the implications of examining the extreme tails of return distributions [21], [22], [28], [29]. In this spirit, we examine volatility return premia by focusing on extreme tails of prices [11].

Table 1 reports statistics that summarize our sample, which consists of much of the universe of securities listed on CRSP. We note that CRSP contains the universe of publicly traded securities that are listed on major stock exchanges in the United States, such as the NYSE, AMEX, and the Nasdaq stock exchange. The CRSP data does not include securities that trade on Over-the-Counter (OTC) markets or the PinkSheets markets. Therefore, the construction of our sample begins with the universe of securities that are listed on the U.S. major exchanges. Some CRSP securities do not have available Compustat data (and visa versa). Therefore, we lose approximately 25% of observations when merging the two datasets together. Panel A shows the distribution of the main variables that we use throughout our analysis. We find that the average stock has a *Ln(Price Range)* of 0.3723. We note that the skewness and kurtosis of the variable are relatively small, which supports the previous arguments [11] that this measure of range-based volatility is approaching a Gaussian distribution. This is particularly true when comparing the distribution of *Ln(Price Range)* to the distribution of *IdioVolt*. For instance, the average stock has an *IdioVolt* of 0.0334, but the variable is heavily (positively) skewed and has excess kurtosis. While the distribution of *Beta* is centered on the mean, the distribution contains a high level of kurtosis. In columns 4 through 7, we find that the average stock in our sample has a market capitalization of $1.71 billion, a book-to-market ratio of 0.4276, momentum of 0.1502, and illiquidity of 9.4178. These summary statistics are similar to those in prior studies in the asset pricing literature.

**Table 1:** Summary statistics and correlation.

Panel A. Summary Statistics							
	*Ln(Price Range)*	*IdioVolt*	*Beta*	*Size*	*B/M*	*Momentum*	*Illiquidity*
	1	2	3	4	5	6	7
Mean	0.3723	0.0334	0.8511	1.7099	0.4276	0.1502	9.4178
Median	0.4447	0.0262	0.8368	0.1268	0.0657	0.1243	0.9991
Std. Deviation	1.1374	0.0262	0.9350	9.9758	11.9400	0.5640	660.29
Skewness	-0.3430	5.5118	-0.0391	19.4530	132.0139	1.9787	1,158.30
Kurtosis	1.10	136.83	16.26	563.25	22,603.43	31.59	1,489,774.96
Panel B. Correlation Matrix							
*Ln(Price Range)*	1.0000	-0.3334	0.2044	0.1678	0.0103	0.2092	-0.0238
		[<.0001]	[<.0001]	[<.0001]	[<.0001]	[<.0001]	[<.0001]
*IdioVolt*		1.0000	-0.0015	-0.1100	-0.0053	-0.0546	0.0498
			[0.0399]	[<.0001]	[<.0001]	[<.0001]	[<.0001]
*Beta*			1.0000	0.0269	-0.0071	0.0778	-0.0102
				[<.0001]	[<.0001]	[<.0001]	[<.0001]
*Size*				1.0000	-0.0052	0.0126	-0.0024
					[<.0001]	[<.0001]	[0.0009]
*B/M*					1.0000	-0.0062	0.0009
						[<.0001]	[0.2215]
*Momentum*						1.0000	-0.0101
							[<.0001]
*Illiquidity*							1.0000

The table reports statistics that describe our sample. Panel A presents some summary statistics for the variables used throughout the analysis. Panel B presents a correlation matrix along with corresponding p-values in brackets. *Ln(Price Range)* is the natural log of the difference between the highest price during a particular month and the lowest price. *IdioVolt* is the idiosyncratic volatility and is obtained by calculating the standard deviation of daily residual returns, where residuals are obtained from a daily four-factor model. *Beta* is the slope coefficient from estimating a daily CAPM. We note that *IdioVolt* and *Beta* are calculated for each stock in each month using a rolling six-month window. *Size* is the market capitalization on the last day of each month in $ Billions. *B/M* is the book-to-market ratio. *Momentum* is the cumulative returns from month t-12 to t-2. *Illiquidity* is the monthly average of the ratio of the absolute value of the daily return scaled by the daily volume (in $ millions).

**Fig 1:**
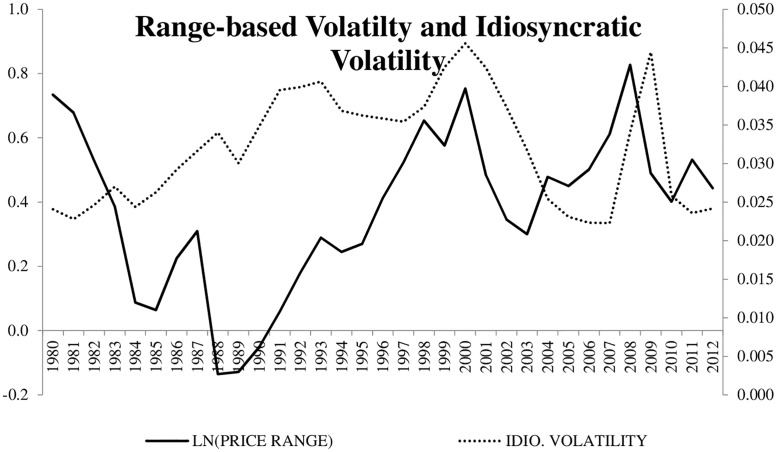
LN(PRICE RANGE) and IDIO. VOLATILITY across the sample time period. The figure shows our measure of Range Based Volatility (Ln(Price Range)) and Idiosyncratic Volatility (Idio. Volatility), which is the standard deviation of daily residual returns that are obtained from a standard four-factor model, for each year in our sample time period.

**Fig 2:**
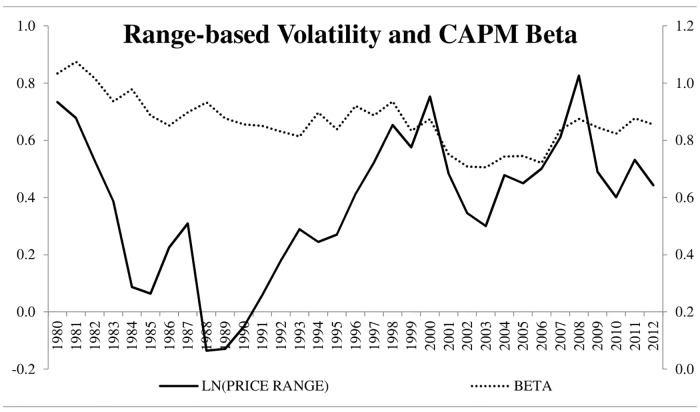
LN(PRICE RANGE) and BETA across the sample time period. The figure shows our measure of Range Based Volatility (Ln(Price Range)) and the CAPM Beta for each year in our sample time period.

Panel B shows a correlation matrix of the variables used throughout the analysis. A few results are noteworthy. We find that *Ln(Price Range)* while positively related to *Beta*, is negatively correlated with *IdioVolt*. These results suggest that range-based volatility is capturing something different than what traditional measures of idiosyncratic volatility capture. The arguments presented in Alizadeh, Brandt, and Diebold [11] suggest that this measure of range-based volatility properly captures stochastic volatility whereas idiosyncratic volatility may be more persistent. We note that estimate idiosyncratic skewness and idiosyncratic kurtosis using daily residual returns (for a six-month window) from Eq (1). We then estimate correlation coefficients between our variable of interest (Ln(Price Range)) and both idiosyncratic skewness and idiosyncratic kurtosis. We find that the negative correlation between *Ln(Price Range)* and skewness is -0.087 while the negative correlation between *Ln(Price Range)* and kurtosis is -0.095, respectively. It is possible that the negative correlation between range-based volatility and idiosyncratic volatility is due to the potential persistence in the latter. Perhaps an investigation that compares range-based (stochastic) volatility to other traditional measures of volatility with built-in persistence may be a fruitful avenue for future research. To continue our summary of the data, we plot range-based volatility and idiosyncratic volatility across are sample time period (Fig 1). Here, we see that the range-based volatility for the average stock seems to be negatively related to the average stock’s idiosyncratic volatility for the first part of our sample time period. When examining the relation between range-based volatility and CAPM beta across time, we do not find a meaningful pattern (Fig 2). In panel B, we also find that *Ln(Price Range)* is positively correlated with *Size*, *B/M*, and *Momentum* and negatively associated with *Illiquidity*. We note that, while significant (due to the number of observations in our sample), the correlation coefficients are relatively close to zero for *B/M* and *Illiquidity*.

## Empirical tests and results

### Range-based volatility and returns—Portfolio analysis

To test for a return premium associated with range-based volatility, we first begin by examining the returns and alphas to value-weighted portfolios that are sorted by *Ln(Price Range)*. Given that prior research [11] finds that the *Ln(Price Range)* is a more efficient estimate of volatility than previously used measures, our hope is that examining range-based volatility might shed some light on the conflicting findings regarding the lead-lag relationship between stock returns and volatility from previous research. Panel A of Table 2 presents the mean (next-month) returns and market-adjusted returns for portfolios sorted on the *Ln(Price Range)*. Market-adjusted returns are the returns in month *t+1* less the value-weighted CRSP market index in month *t+1*. We note that the CRSP value-weighted market index consists of the weighted average of all U.S. stocks listed on CRSP, where weights are based on the market capitalization of each stock. Column 1 details the portfolio returns from the lowest quintile of *Ln(Price Range)*. Columns 2 through 5 provide the findings from quintiles 2 through 5 respectively. In column 6, we report the difference between extreme portfolios (Q5 –Q1) along with corresponding *t*-statistics that test whether the high minus low returns are significantly different from zero. For both rows in Panel A, we find mean returns and market-adjusted returns are decreasing monotonically across increasing *Ln(Price Range)* portfolios. The Q5 –Q1 difference in mean returns is -0.0114 (*t*-statistic = -4.15). Similar results are found when looking at adjusted returns. These high minus low differences are not only statistically significant but they are also economically meaningful. For example, in annual terms, the difference in raw returns is more than 13.5%. The results in Panel A corroborate previous findings [7], [8], [30] and necessitate further examination. We recognize, however, the need to control for other risk factors in a more multivariate setting.

**Table 2:** Portfolio analysis.

Panel A. Mean Returns across Value-Weighted Portfolios						
	Q1 (Low)	Q2	Q3	Q4	Q5 (High)	Q5 –Q1
	1	2	3	4	5	6
Mean Returns	0.0213	0.0123	0.0105	0.0104	0.0099	-0.0114***
						(-4.15)
Adj. Returns	0.0191	0.0101	0.0083	0.0082	0.0078	-0.0113***
						(-4.15)
Panel B. CAPM Regressions by Value-Weighted Portfolios						
Alpha	0.0114***	0.0023	0.0004	-0.0001	-0.0013	-0.0127***
	(4.19)	(1.25)	(0.32)	(-0.05)	(-1.17)	(-4.32)
*MKT*	0.9821***	1.0024***	1.0143***	1.0800***	1.2141***	0.2320***
	(16.08)	(19.91)	(26.51)	(38.63)	(47.87)	(3.51)
Panel C. Fama and French [9] Multifactor Regressions by Value-Weighted Portfolios						
Alpha	0.0092***	0.0001	-0.0014	-0.0013**	-0.0010	-0.0102***
	(3.71)	(0.07)	(-1.58)	(-2.28)	(-1.58)	(-3.98)
*MKT*	0.9184***	0.9655***	0.9842***	1.0392***	1.0946***	0.1762**
	(13.29)	(24.88)	(39.61)	(68.47)	(64.46)	(2.48)
*HML*	0.3849***	0.3999***	0.3269***	0.2141***	-0.1217***	-0.5066***
	(2.89)	(5.24)	(6.95)	(7.48)	(-4.65)	(-3.74)
*SMB*	1.0222***	0.8685***	0.7096***	0.6036***	0.5960***	-0.4262***
	(8.02)	(9.43)	(12.65)	(15.75)	(18.04)	(-3.24)
Panel D. Carhart [12] Multifactor Regressions by Value-Weighted Portfolios						
Alpha	0.0129***	0.0028*	0.0005	-0.0004	-0.0012*	-0.0141***
	(4.89)	(1.87)	(0.54)	(-0.78)	(-1.91)	(-5.20)
*MKT*	0.8190***	0.8916***	0.9349***	1.0157***	1.1010***	0.2820***
	(12.00)	(23.09)	(39.79)	(68.95)	(65.05)	(4.01)
*HML*	0.2288*	0.2839***	0.2494***	0.1772***	-0.1118***	-0.3406***
	(1.76)	(4.01)	(5.89)	(6.53)	(-4.41)	(-2.57)
*SMB*	1.0324***	0.8761***	0.7147***	0.6060***	-0.5954***	-1.6278***
	(10.26)	(12.89)	(17.54)	(18.91)	(18.92)	(-15.44)
*UMD*	-0.4156***	-0.3089***	-0.2062***	-0.0982***	0.0265	0.4421***
	(-4.68)	(-4.89)	(-5.64)	(-5.22)	(1.41)	(4.87)

The table report returns and alphas across value-weighted portfolios sorted by *Ln(Price Range)*, which is the natural log of the difference between the highest price during a particular month and the lowest price. Panel A presents the Mean Returns and Adj. Returns. We note that Adj. Returns are returns in month t+1 less the value-weighted CRSP market index. Column 6 reports the difference between extreme portfolios along with corresponding t-statistics. Panels B through D present the results from estimating variants of the following equation using 384 months of data by value-weighted portfolios based on the *Ln(Price Range)*.

$R_{p,t+1}-\;R_{f,t+1}=\alpha +\beta_{MKT}MKT_{t+1}+\beta_{HML}HML_{t+1}+\beta_{SMB}SMB_{t+1}+\beta_{UMD}UMD_{t+1}+\varepsilon_{p,t+1}$ The dependent variable is the excess return of the portfolio over the 1-month T-Bill yield. The independent variable includes MRP, which is the market risk premium, or the excess return of the market less the risk-free rate. *SMB* is the small-minus-big return factor while *HML* is the high-minus-low return factor. *UMD* is the up-minus-down factor. The dependent and independent variables are measured over month t+1 while the portfolios are sorted at the end of month t. Panel B shows the results for CAPM regressions. Panel C presents the findings for the three-factor regressions. Panel D shows the results from the full specification. Robust t-statistics are reported in parentheses.

*denote statistical significance at the 0.10 level.

** denote statistical significance at the 0.05 level.

*** denote statistical significance at the 0.01 levels.

Panels B through D of Table 2 present the results from estimating variants of the following equation using 384 months of value-weighted portfolios based on *Ln(Price Range)*.

$$R_{p,t+1}-\;R_{f,t+1}=\alpha +\beta_{MKT}MKT_{t+1}+\beta_{HML}HML_{t+1}+\beta_{SMB}SMB_{t+1}+\beta_{UMD}UMD_{t+1}+\varepsilon_{p,t+1}$$ (2)

Eq (2) is identical to Eq (1) with three exceptions. First, we estimate the equation for each portfolio *p* instead of each stock *i*. Second, the subscript *t* represents months instead of days during our sample time period. Third, the dependent and independent variables are measured over month *t+1* while the portfolios are sorted at the end of month *t*. As before, the dependent variable is the *monthly* excess return of the portfolio over the one-month yield on T-Bills. The independent variables are the various monthly risk factors. Panel B shows the results for CAPM regressions (i.e., we restrict *β*_*HML*_ *= β*_*SMB*_ *= β*_*UMD*_ = 0). Panel C presents the findings for the three-factor regressions (i.e., we restrict *β*_*UMD*_ = 0). Panel D shows the results from the full specification. Robust *t*-statistics are reported in parentheses [31].

For brevity, in the discussion of Table 2, we focus primarily on the alphas from estimating Eq (2) although we also report the coefficients on the various risk factors. Panel B reports the results from a CAPM regression that uses the CRSP value-weighted portfolio as the market return. Only in the low *Ln(Price Range)* portfolio do we find a positive and significant alpha. Further, alphas are monotonically decreasing across increasing portfolios of *Ln(Price Range)*. The difference between extreme portfolios is again negative and significant (difference = -0.0127, *t*-statistic = -4.32), supporting our findings in Panel A and suggesting that *Ln(Price Range)* is associated with a negative return premium. Panel C estimates alphas using a Fama and French (1993 and 1996) three-factor model. Again we find that alphas are generally decreasing, although not monotonically. However, the difference between Q5 and Q1 is -0.0102 (*t*-statistic = -3.98), suggesting that, after holding the three risk factors constant, the return premium associated with *Ln(Price Range)* is about 1% per month. Qualitatively similar results are found in Panel D where we estimate alphas using a four-factor model [12]. Here, we find that alphas are again decreasing monotonically across portfolios. Further, we find that, in the highest portfolio, the alpha is negative and marginally significant (estimate = -0.0012, *t*-statistic = -1.91). As before, the difference between extreme portfolios is negative and statistically significant (difference = -0.0141, *t*-statistic = -5.20).

Although these time-series regressions indicate that *Ln(Price Range)* is associated with a significantly lower stock returns, prior research has shown that a number of stock characteristics can influence the cross-section of stock returns. In the next section, we examine the cross-sectional relationship between *Ln(Price Range)* and next-month returns using Fama-MacBeth regressions [13].

### Range-based volatility and returns—A Fama and MacBeth approach

Table 3 presents results from estimating the following equation using pooled stock-month data. We note that we estimate 384 cross-sectional regressions using a Fama-MacBeth approach [13].

$$\begin{array}{c} R_{i,t+1}=\beta_{1}Ln(Price\;Range_{i,t})+\beta_{2}Beta_{i,t}+\beta_{3}Size_{i,t}+\beta_{4}B/M_{i,t}+\beta_{5}Momentum_{i,t}+\\ \beta_{6}Illiquidity_{i,t}+\alpha +\varepsilon_{i,t+1} \end{array}$$ (3)

**Table 3:** Fama-MacBeth regressions.

	Partial Specifications						Full Specification
Specification:	1	2	3	4	5	6	7
*Ln(Price Range)*	-0.3928***	-0.3781***	-0.2436***	-0.3180***	-0.4196***	-0.3510***	-0.3066***
	(-4.05)	(-3.73)	(-2.69)	(-3.43)	(-4.57)	(-3.68)	(-3.97)
*Beta*		-0.0674					-0.0282
		(-0.76)					(-0.33)
*Size*			-0.1130***				-0.0115
			(-2.61)				(-0.24)
*B/M*				0.6312***			0.6420***
				(9.13)			(9.69)
*Momentum*					0.2816*		0.5636***
					(1.67)		(3.84)
*Illiquidity*						0.0093***	0.0079***
						(3.16)	(3.13)
*Constant*	1.4481***	1.4883***	2.7120***	3.1181***	1.3523***	1.4002***	3.0808***
	(4.07)	(4.75)	(3.71)	(8.62)	(3.99)	(3.97)	(4.79)

The table reports the results from estimating variants of the following equation using a Fama-MacBeth regression.

$$R_{i,t+1}=\beta_{1}Ln(Price\;Range_{i,t})+\beta_{2}Beta_{i,t}+\beta_{3}Size_{i,t}+\beta_{4}B/M_{i,t}+\beta_{5}Momentum_{i,t}+\beta_{6}Illiquidity_{i,t}+\alpha +\varepsilon_{i,t+1}$$ The dependent variable is the monthly return for stock *i* in month *t+1*. The independent variable of interest is *Ln(Price Range)*, which is the natural log of the difference between the highest price during a particular month and the lowest price. The control variables include the following. *Beta* is the CAPM beta obtained from estimating a standard daily CAPM data using a six-month rolling window. *Size* is the natural log of end-of-month market capitalization (in $Billions). *B/M* is the natural log of the book-to-market ratio for each stock in each month. *Momentum* is the cumulative return from month *t-12* to *t-2*. *Illiquidity* is the monthly average of the ratio of the absolute value of the daily return scaled by dollar volume (in $Millions). In parenthesis, we report t-statistics that are obtained from adjusted standard errors that account for three lags.

*denotes statistical significance at the 0.10 level.

** denotes statistical significance at the 0.05 level.

*** denotes statistical significance at the 0.01 level.

The dependent variable is the monthly return for stock *i* in month *t+1*. The independent variable of interest is *Ln(Price Range)*, which is the natural log of the difference between the highest price during a particular month and the lowest price. The control variables include the following. *Beta*, which is the CAPM beta obtained from estimating a standard daily market model using a six-month rolling window. *Size* is the natural log of end-of-month market capitalization (in $Billions). *B/M* is the natural log of the book-to-market ratio for each stock in each month. *Momentum* is the cumulative return from month *t-12* to *t-2*. *Illiquidity* is the Amihud (2002) measure of illiquidity, which is the average daily ratio of the absolute value of the return scaled by dollar volume (in $Millions). In parenthesis, we report *t*-statistics that are obtained from standard errors that account for three lags [32].

Column 1 of Table 3 is a simple regression that only includes the *Ln(Price Range)* as an independent variable. Similar to our previously documented results, *Ln(Price Range)* is negatively related to next month returns and is highly significant (estimate = -0.3928, *t*-statistic = -4.05). Columns 2 through 6 of Table 3 replicate Column 1 but include each of the control variables separately. The included control variables have the expected signs. *Beta* is positive but insignificant, *Size* is negative and significant and has the greatest impact on the estimation of the coefficient on *Ln(Price Range)*. The remaining control variables, *B/M*, *Momentum*, and *Illiquidity* are each positively related to next-month returns and vary in their significance. Although including the individual control variables does have some impact on the estimated effect of *Ln(Price Range)*, the coefficient remains highly statistically significant in each of the first six columns. Column 7 of Table 3 reports the results from our full specification. Here, we again find that *Ln(Price Range)* produces a negative and significant estimate (estimate = -0.3066, *t*-statistic = -3.97). The coefficient is not only statistically significant, but it is also economically meaningful as a one standard deviation increase in *Ln(Price Range)* corresponds to a 35 basis point reduction in next-month returns. Thus, these cross-sectional regression results support our findings in the previous table and suggest that range-based volatility is negatively associated with expected returns.

Given that several papers [7], [8] have documented a negative return premium associated with idiosyncratic volatility, it might be important to add *IdioVolt* as an additional control variable. Next, we estimate the following equation.

$$\begin{array}{c} R_{i,t+1}=\beta_{1}Ln(Price\;Range_{i,t})+\beta_{2}IdioVolt_{i,t}+\beta_{3}Beta_{i,t}+\beta_{4}Size_{i,t}+\beta_{5}B/M_{i,t}+\beta_{6}Momentum_{i,t}+\\ \beta_{7}Illiquidity_{i,t}+\alpha +\varepsilon_{i,t+1} \end{array}$$ (4)

In Eq (4), the dependent and independent variables are identical to those in Eq (3) with one exception. Here, we include *IdioVolt* as an additional control variable. As before, we estimate Eq (4) using a Fama-MacBeth approach [13]. We control for heteroskedasticity and autocorrelation in error terms and report *t*-statistics from standard errors that have been corrected with three lags [32]. We report these results in Table 4.

**Table 4:** Fama-MacBeth regressions.

	Partial Specifications								Full Specification
Specification:	1	2	3	4	5	6	7	8	9
*Ln(Price Range)*			-0.3388***	-0.3214***	-0.2238***	-0.2385***	-0.3821***	-0.3201***	-0.2954***
			(-4.31)	(-4.06)	(-2.80)	(-3.30)	(-5.26)	(-4.11)	(-4.12)
*IdioVolt*	2.7090	-0.1560	-3.2780	-2.4734	-7.6437	1.0961	-3.6674	-5.7789	-1.4156
	(0.45)	(-0.03)	(-0.56)	(-0.43)	(-1.29)	(0.20)	(-0.65)	(-0.96)	(-0.27)
*Beta*		-0.0361		-0.0428					-0.0145
		(-0.48)		(-0.53)					(-0.19)
*Size*		-0.0746**			-0.1153***				0.0199
		(-2.15)			(-4.02)				(0.60)
*B/M*		0.6490***				0.6136***			0.6468***
		(11.33)				(10.36)			(11.21)
*Momentum*		0.5386***					0.3622**		0.6187***
		(3.86)					(2.39)		(4.61)
*Illiquidity*		0.0082***						0.0097***	0.0081***
		(3.30)						(3.37)	(3.24)
*Constant*	1.0444***	3.6392***	1.3991***	1.3827***	2.8399***	2.8935***	1.3136***	1.4416***	2.6348***
	(4.37)	(7.47)	(5.38)	(5.97)	(6.60)	(9.54)	(5.26)	(5.59)	(6.26)

The table reports the results from estimating variants of the following equation using a Fama-MacBeth (1973) regression.

$$R_{i,t+1}=\beta_{1}Ln(Price\;Range_{i,t})+\beta_{2}IdioVolt_{i,t}+\beta_{3}Beta_{i,t}+\beta_{4}Size_{i,t}+\beta_{5}B/M_{i,t}+\beta_{6}Momentum_{i,t}+\beta_{7}Illiquidity_{i,t}+\alpha +\varepsilon_{i,t+1}$$ The dependent variable is the monthly return for stock *i* in month *t+1*. The independent variables of interest are *Ln(Price Range)*, which is the natural log of the difference between the highest price during a particular month and the lowest price, and *IdioVolt*, which is obtained by calculating the standard deviation of daily residual returns, where residuals are obtained from a daily four-factor model. The control variables include the following. *Beta* is the CAPM beta obtained from estimating a standard daily CAPM data using a six-month rolling window. *Size* is the natural log of end-of-month market capitalization (in $Billions). *B/M* is the natural log of the book-to-market ratio for each stock in each month. *Momentum* is the cumulative return from month *t-12* to *t-2*. *Illiquidity* is the monthly average of the ratio of the absolute value of the daily return scaled by dollar volume (in $Millions). In parenthesis, we report t-statistics that are obtained from adjusted standard errors that account for three lags.

*denote statistical significance at the 0.10 level.

** denote statistical significance at the 0.05 level.

*** denote statistical significance at the 0.01 levels.

Column 1 presents the results from a simple regression where the only independent variable is *IdioVolt*. Results show that the estimate for *IdioVolt* is not reliably different from zero. Column 2 shows that when controlling for the other variables (except *Ln(Price Range)*), the coefficient on *IdioVolt* is again statistically close to zero. These findings differ from Ang et al. [8] that show a reliably negative coefficient for their measure of idiosyncratic volatility. Perhaps the reason for this discrepancy is due to the different ways in which our measure of idiosyncratic volatility is calculated. Ang et al. [8] estimate idiosyncratic volatility using the daily residuals from a three-factor model instead of a four-factor model. Not finding a reliable estimate for idiosyncratic volatility is not surprising, given that results in Fu [10] show the relationship between idiosyncratic volatility and next-month returns is fragile and heavily depends on how volatility is estimated. At a very minimum, the culmination of prior work indicates that the cross-sectional relationship between idiosyncratic volatility and next-month returns is not very robust to differences in the way volatility is calculated.

Columns 3 through 9 replicate the analysis in the previous table but include both *IdioVolt* and *Ln(Price Range)* in each of the specifications. The most important result is that *Ln(Price Range)* produces a negative estimate that is both statistically and economically significant in each of the specifications. Further, *IdioVolt* does not produce a reliable coefficient in any of the specifications. In the full specification, we find that, while *B/M*, *Momentum*, and *Illiquidity* are positively related to next-month returns, *Ln(Price Range)* still produces an estimate of -0.2954 (*t*-statistic = -4.12). In economic terms, the results in column 9 suggest that, after holding other variables constant, a one standard deviation increase in *Ln(Price Range)* is associated with a 34 basis point reduction in next-month returns. Combined with findings in the previous two tables, these tests reveal a reliable, negative return premium associated with range-based volatility.

### Do traditional measures of risk explain the range-based volatility return premium

In this section, we attempt to explain which factors drive the return premium associated with range-based volatility. In our first of two sets of tests, we create subsamples based on other common measures of risk, idiosyncratic volatility, and beta, and then estimate Eq (3) using the Fama-Macbeth [13] approach for each of the subsamples. We begin by sorting stocks into terciles based on *IdioVolt* during each month of our sample time period. We then test whether the return premium associated with *Ln(Price Range)* is strongest in the high idiosyncratic volatility tercile. Table 5 reports the results of this analysis.

**Table 5:** Fama-MacBeth regressions on idiosyncratic volatility terciles.

	Low *IdioVolt*	Mid *IdioVolt*	High *IdioVolt*
	1	2	3
*Ln(Price Range)*	-0.0599	-0.1891***	-0.3702***
	(-1.56)	(-2.75)	(-3.17)
*Beta*	0.0174	0.0107	0.0167
	(0.14)	(0.11)	(0.27)
*Size*	-0.0220	0.0257	-0.4502***
	(-0.78)	(0.68)	(-5.91)
*B/M*	0.1183***	0.5037***	1.1963***
	(3.08)	(8.51)	(13.17)
*Momentum*	1.0314***	1.2490***	0.5732***
	(4.49)	(7.32)	(4.58)
*Illiquidity*	-0.0018	-0.0135*	0.0048**
	(-0.06)	(-1.87)	(2.34)
*Constant*	1.6203***	2.0724***	8.8785***
	(3.96)	(4.65)	(10.73)

The table reports the results from estimating variants of the following equation using a Fama-MacBeth (1973) regression for three subsamples.

$$\begin{array}{c} R_{i,t+1}=\beta_{1}Ln(Price\;Range_{i,t})+\beta_{2}IdioVolt_{i,t}+\beta_{3}Beta_{i,t}+\beta_{4}Size_{i,t}+\beta_{5}B/M_{i,t}+\beta_{6}Momentum_{i,t}+\\ \beta_{7}Illiquidity_{i,t}+\alpha +\varepsilon_{i,t+1} \end{array}$$ The dependent variable is the monthly return for stock *i* in month *t+1*. The independent variables of interest are *Ln(Price Range)*, which is the natural log of the difference between the highest price during a particular month and the lowest price, and *IdioVolt*, which is obtained by calculating the standard deviation of daily residual returns, where residuals are obtained from a daily four-factor model. The control variables include the following. *Beta* is the CAPM beta obtained from estimating a standard daily CAPM data using a six-month rolling window. *Size* is the natural log of end-of-month market capitalization (in $Billions). *B/M* is the natural log of the book-to-market ratio for each stock in each month. *Momentum* is the cumulative return from month *t-12* to *t-2*. *Illiquidity* is the monthly average of the ratio of the absolute value of the daily return scaled by dollar volume (in $Millions). In each month, we sort stocks into terciles based on *IdioVolt*. Column 1 reports the results for the bottom tercile. Columns 2 and 3 present the results for the middle and top terciles, respectively. In parenthesis, we report t-statistics that are obtained from adjusted standard errors that account for three lags.

* denote statistical significance at the 0.10 level.

** denote statistical significance at the 0.05 level.

*** denote statistical significance at the 0.01 level.

For brevity, we focus our discussion on the coefficients for *Ln(Price Range)* in this table and those that follow. The first row of Table 5 shows that the estimates for *Ln(Price Range)* are decreasing monotonically across the increasing terciles. In column 1, we do not find a significant return premium associated with *Ln(Price Range)* as the coefficient is -0.0599 and the corresponding *t*-statistic is -1.56. We do, however, find that in the Mid *IdioVolt* tercile, the estimate for *Ln(Price Range)* is negative and statistically significant (estimate = -0.1891, *t*-statistic = -2.75). Finally, in the highest *IdioVolt* tercile, the coefficient of interest is -0.3702 (t-statistic = -3.17). The difference in the coefficient between columns 1 and 3 is statistically significant (z-statistic = 2.54) suggesting that the return premium is significantly stronger in the high idiosyncratic volatility tercile compared to the low idiosyncratic volatility tercile. In an additional comparison, the estimate for *Ln(Price Range)* in column 3 is slightly more than 20% larger (in absolute value) than the corresponding coefficient in column 7 of Table 3, suggesting that idiosyncratic volatility indeed drives the range-based volatility return premium.

Next, we continue our analysis by determining whether the return premium is stronger in high beta stocks. As before, we sort stocks into terciles based on our estimates of beta. We then estimate Eq (3) for each subsample using a Fama-MacBeth approach [13], which is presented in Table 6. A few results are noteworthy. First, while the coefficients on *Ln(Price Range)* are reliably negative across each of the columns, the estimate is most negative in column 3, the high beta tercile. We note, however, that the coefficient is not monotonically decreasing across increasing beta terciles. Further, while the coefficient on *Ln(Price Range)* decreases approximately 21% from column 1 to column 3, the difference between coefficients in these two columns is not statistically significant (z-statistic = 0.49). Therefore, in Table 6, we only find weak evidence that the return premium associated with range-based volatility is driven by high beta stocks.

**Table 6:** Fama-MacBeth regressions on beta terciles.

	Low *Beta*	Mid *Beta*	High *Beta*
	1	2	3
*Ln(Price Range)*	-0.2967***	-0.2475***	-0.3581***
	(-3.83)	(-3.56)	(-3.57)
*Beta*	-0.1103	0.1274	-0.2376**
	(-0.86)	(0.68)	(-2.01)
*Size*	-0.0723	0.0013	-0.0080
	(-1.55)	(0.03)	(-0.15)
*B/M*	0.6923***	0.4680***	0.8077***
	(12.03)	(7.29)	(9.14)
*Momentum*	0.5366***	0.5395***	0.7053***
	(3.30)	(2.94)	(4.41)
*Illiquidity*	0.0077***	0.0069	0.0128
	(3.71)	(0.95)	(1.45)
*Constant*	3.7261***	2.3321***	3.9227***
	(6.05)	(3.65)	(5.93)

The table reports the results from estimating variants of the following equation using a Fama-MacBeth (1973) regression for three subsamples.

$$\begin{array}{c} R_{i,t+1}=\beta_{1}Ln(Price\;Range_{i,t})+\beta_{2}IdioVolt_{i,t}+\beta_{3}Beta_{i,t}+\beta_{4}Size_{i,t}+\beta_{5}B/M_{i,t}+\beta_{6}Momentum_{i,t}+\\ \beta_{7}Illiquidity_{i,t}+\alpha +\varepsilon_{i,t+1} \end{array}$$ The dependent variable is the monthly return for stock *i* in month *t+1*. The independent variables of interest are *Ln(Price Range)*, which is the natural log of the difference between the highest price during a particular month and the lowest price, and *IdioVolt*, which is obtained by calculating the standard deviation of daily residual returns, where residuals are obtained from a daily four-factor model. The control variables include the following. *Beta* is the CAPM beta obtained from estimating a standard daily CAPM data using a six-month rolling window. *Size* is the natural log of end-of-month market capitalization (in $Billions). *B/M* is the natural log of the book-to-market ratio for each stock in each month. *Momentum* is the cumulative return from month *t-12* to *t-2*. *Illiquidity* is the monthly average of the ratio of the absolute value of the daily return scaled by dollar volume (in $Millions). In each month, we sort stocks into terciles based on *Beta*. Column 1 reports the results for the bottom tercile. Columns 2 and 3 present the results for middle and top terciles, respectively. In parenthesis, we report t-statistics that are obtained from adjusted standard errors that account for three lags.

* denote statistical significance at the 0.10 level.

** denote statistical significance at the 0.05 level.

*** denote statistical significance at the 0.01 level.

In unreported tests, we include *IdioVolt* as an additional control variable and we are able to draw similar conclusions to those drawn in Tables 5 and 6. As additional robustness tests, we also sort stocks into quintiles instead of terciles based on both idiosyncratic volatility and beta. Again, we find qualitatively similar results to those reported in these two tables. Combined, the results suggest that the negative return premium associated with range-based volatility is driven by stocks with high idiosyncratic volatility and, to a lesser extent, stocks with high beta.

### Lottery stocks and the range-based volatility return premium

In this subsection, we continue to explore factors that influence the return premium associated with range-based volatility. In the previous subsection, we find that other traditional measures of risk can partially explain the observed return premium. We note that prior literature has found a significantly negative cross-sectional relationship between either idiosyncratic volatility or beta and future returns [7], [8], [30]. Some researchers [25], [26] have argued that preferences for riskier stocks, which results in demand-induced price premiums and subsequent underperformance of such stocks, may indeed be related to preferences for stocks that resemble lotteries. For instance, research in Kumar [25] argues that, among other characteristics, higher levels of idiosyncratic volatility may contribute to the resemblance of lotteries. In particular, he suggests that stocks with high idiosyncratic volatility, high idiosyncratic skewness, and low stock prices are more likely to resemble lottery-like payoffs and classifies such stocks as lottery stocks. In his analysis, he finds that lottery stocks significantly underperform non-lottery stocks indicating an unusual level of demand for these stocks. Similarly, Bali, et al. [26] find that the return premium for either beta or idiosyncratic volatility becomes negligible when controlling for lottery demand. If the preference for higher risk stocks is simply a subset for a larger preference for lottery-like stocks, then the return premium associated with range-based volatility should be driven by these types of stocks.

In the next three tables, we replicate the analysis in Tables 5 and 6 but instead of creating subsamples of stocks based on risk, we create subsamples based on lottery-type stocks. We first estimate Eq (3) for stocks that are classified as lottery stocks [25] and those that are not. In particular, we denote a stock to be a lottery stock if, during a particular time period, the stock has idiosyncratic volatility above the median, idiosyncratic skewness above the median, and a share price below the median. We note that idiosyncratic skewness is estimated similarly to idiosyncratic volatility except we calculate the skewness of daily residual returns (where residuals come from a daily four-factor model) instead of the standard deviation of residual returns. As before, we use a rolling six-month period in order to allow for a proper number of observations for the sake of better accuracy when estimating moments of the return distribution. Approximately 20% of stocks are classified as lottery stocks according to this definition, which is similar to prior research [25].

Table 7 presents the analysis. Column 1 shows the results for the subsample of stocks that are classified as lottery stocks while column 2 presents the results for non-lottery stocks. Interestingly, we find that the coefficient on *Ln(Price Range)* is -0.5552 (t-statistic = -4.55) in column 1 and -0.2309 (t-statistic = -3.69) in column 2. Not only is the coefficient of interest more than twice as negative in column 1 than in column 2, but the difference between coefficients is also statistically significant (z-statistic = 2.36). These results suggest that the return premium associated with range-based volatility is stronger for stocks that resemble lotteries than for stocks that do not.

**Table 7:** Fama-MacBeth regressions on lottery stocks.

	Lottery Stocks	Non-Lottery Stocks
	1	2
*Ln(Price Range)*	-0.5552***	-0.2309***
	(-4.55)	(-3.69)
*Beta*	0.0374	0.0088
	(0.55)	(0.08)
*Size*	-0.3506***	-0.0155
	(-4.53)	(-0.39)
*B/M*	1.2110***	0.4370***
	(12.61)	(8.08)
*Momentum*	0.6791***	0.5931***
	(5.02)	(3.38)
*Illiquidity*	0.0052	0.0100***
	(1.43)	(3.39)
*Constant*	7.7785***	2.5112***
	(9.10)	(4.58)

The table reports the results from estimating variants of the following equation using a Fama-MacBeth (1973) regression for three subsamples.

$$\begin{array}{c} R_{i,t+1}=\beta_{1}Ln(Price\;Range_{i,t})+\beta_{2}IdioVolt_{i,t}+\beta_{3}Beta_{i,t}+\beta_{4}Size_{i,t}+\beta_{5}B/M_{i,t}+\beta_{6}Momentum_{i,t}+\\ \beta_{7}Illiquidity_{i,t}+\alpha +\varepsilon_{i,t+1} \end{array}$$ The dependent variable is the monthly return for stock *i* in month *t+1*. The independent variables of interest are *Ln(Price Range)*, which is the natural log of the difference between the highest price during a particular month and the lowest price, and *IdioVolt*, which is obtained by calculating the standard deviation of daily residual returns, where residuals are obtained from a daily four-factor model. The control variables include the following. *Beta* is the CAPM beta obtained from estimating a standard daily CAPM data using a six-month rolling window. *Size* is the natural log of end-of-month market capitalization (in $Billions). *B/M* is the natural log of the book-to-market ratio for each stock in each month. *Momentum* is the cumulative return from month *t-12* to *t-2*. *Illiquidity* is the monthly average of the ratio of the absolute value of the daily return scaled by dollar volume (in $Millions). In each month, we classify stocks as lottery or non-lottery stocks. Column 1 reports the results for lottery stocks and column 2 reports the results for non-lottery stocks. In parenthesis, we report t-statistics that are obtained from adjusted standard errors that account for three lags.

* denote statistical significance at the 0.10 level.

** denote statistical significance at the 0.05 level.

*** denote statistical significance at the 0.01 level.

Next, we sort stocks based on an alternative measure that captures lottery-like stocks. Boyer and Vorkink [21] estimate expected idiosyncratic skewness (*E[IdioSkew]*) using a predictive regression where prior skewness, volatility, momentum, and turnover (among other variables) are used to predict idiosyncratic skewness. They show that stocks with the highest expected idiosyncratic skewness significantly underperform stocks with the lowest expected idiosyncratic skewness suggesting that, again, investor preferences for these types of stocks lead to price premiums and lower future returns. Table 8 reports the results from estimating Eq (3) using regressions for three subsamples of stocks that have been created using expected idiosyncratic skewness [21]. Focusing again on the variable of interest, *Ln(Price Range)*, we find that the coefficient on this variable is decreasing monotonically across increasing *E[IdioSkew]* terciles. The coefficient on *Ln(Price Range)* in the low *E[IdioSkew]* tercile (column 1) is positive but statistically close to zero (estimate = 0.0271, *t-*statistic = 0.39). The same coefficient in the mid *E[IdioSkew]* tercile (column 2) is -0.2012 (*t*-statistic = -2.56). Finally, the coefficient in column 3, the high *E[IdioSkew]* tercile, is -0.3966 (*t*-statistic = -3.34). The z-statistic testing for a significant difference between coefficients in columns 1 and 3 is 3.08 suggesting that stocks with high expected idiosyncratic skewness drive the return premium associated with range-based volatility. These findings corroborate the results in Table 7 that suggest that lottery-type stocks help explain the negative return premium in stocks with high *Ln(Price Range)*.

**Table 8:** Fama-MacBeth regressions on E[IdioSkew] terciles.

	Low *E[IdioSkew]*	Mid *E[IdioSkew]*	High *E[IdioSkew]*
	1	2	3
*Ln(Price Range)*	0.0271	-0.2012**	-0.3966***
	(0.39)	(-2.56)	(-3.34)
*Beta*	-0.0512	-0.1022	0.0962
	(-0.38)	(-0.99)	(1.16)
*Size*	0.0092	0.0315	-0.5112***
	(0.20)	(0.70)	(-5.63)
*B/M*	0.7926***	1.0423***	1.3768***
	(9.22)	(10.08)	(12.02)
*Momentum*	0.7192***	1.0646***	0.5170***
	(3.78)	(6.09)	(3.45)
*Illiquidity*	0.9534	0.0015	0.0051**
	(0.68)	(0.06)	(2.42)
*Constant*	3.2435***	3.6913***	9.8825***
	(4.70)	(5.75)	(10.29)

The table reports the results from estimating variants of the following equation using a Fama-MacBeth (1973) regression for three subsamples.

$$\begin{array}{c} R_{i,t+1}=\beta_{1}Ln(Price\;Range_{i,t})+\beta_{2}IdioVolt_{i,t}+\beta_{3}Beta_{i,t}+\beta_{4}Size_{i,t}+\beta_{5}B/M_{i,t}+\beta_{6}Momentum_{i,t}+\\ \beta_{7}Illiquidity_{i,t}+\alpha +\varepsilon_{i,t+1} \end{array}$$ The dependent variable is the monthly return for stock *i* in month *t+1*. The independent variables of interest are *Ln(Price Range)*, which is the natural log of the difference between the highest price during a particular month and the lowest price, and *IdioVolt*, which is obtained by calculating the standard deviation of daily residual returns, where residuals are obtained from a daily four-factor model. The control variables include the following. *Beta* is the CAPM beta obtained from estimating a standard daily CAPM data using a six-month rolling window. *Size* is the natural log of end-of-month market capitalization (in $Billions). *B/M* is the natural log of the book-to-market ratio for each stock in each month. *Momentum* is the cumulative return from month *t-12* to *t-2*. *Illiquidity* is the monthly average of the ratio of the absolute value of the daily return scaled by dollar volume (in $Millions). In each month, we sort stocks into terciles based on *E[IdioSkew]*. Column 1 reports the results for the bottom tercile. Columns 2 and 3 present the results for middle and top terciles. In parenthesis, we report t-statistics that are obtained from adjusted standard errors that account for three lags.

* denote statistical significance at the 0.10 level.

** denote statistical significance at the 0.05 level.

*** denote statistical significance at the 0.01 level.

In our final set of tests, we continue our analysis by creating subsamples based on the measure of *MaxRet* [22], which is the daily maximum return during a particular month. Bali et al. [22] argue that higher levels of *MaxRet* are an important signal to investors with preferences for lottery-like characteristics. Consistent with the prior work in this area, they show that stocks with the highest *MaxRet* significantly underperform stocks with the lowest *MaxRet*, which again indicates that investors with preferences for lottery-like returns might bid up prices in these particular stocks.

Table 9 reports the results estimating Eq (3) using a Fama-MacBeth approach [13]. Columns 1 through 3 present the regressions by the subsamples based on *MaxRet*.

**Table 9:** Fama-MacBeth regressions on MaxRet terciles.

	Low *MaxRet*	Mid *MaxRet*	High *MaxRet*
	1	2	3
*Ln(Price Range)*	-0.0235	-0.2554***	-0.4059***
	(-0.48)	(-3.10)	(-3.20)
*Beta*	0.1554	0.1132	-0.0601
	(1.24)	(1.17)	(-0.84)
*Size*	-0.0500	-0.0251	-0.1772***
	(-1.42)	(-0.69)	(-2.75)
*B/M*	0.2259***	0.4591***	1.1706***
	(5.16)	(7.27)	(13.44)
*Momentum*	0.5945***	0.9319***	0.6006***
	(3.22)	(5.35)	(4.25)
*Illiquidity*	-0.0122	0.0105*	0.0057***
	(-0.90)	(1.83)	(2.71)
Constant	2.2402***	2.6399***	6.1272***
	(4.40)	(5.39)	(8.03)

The table reports the results from estimating variants of the following equation using a Fama-MacBeth (1973) regression for three subsamples.

$$\begin{array}{c} R_{i,t+1}=\beta_{1}Ln(Price\;Range_{i,t})+\beta_{2}IdioVolt_{i,t}+\beta_{3}Beta_{i,t}+\beta_{4}Size_{i,t}+\beta_{5}B/M_{i,t}+\beta_{6}Momentum_{i,t}+\\ \beta_{7}Illiquidity_{i,t}+\alpha +\varepsilon_{i,t+1} \end{array}$$

The dependent variable is the monthly return for stock *i* in month *t+1*. The independent variables of interest are *Ln(Price Range)*, which is the natural log of the difference between the highest price during a particular month and the lowest price, and *IdioVolt*, which is obtained by calculating the standard deviation of daily residual returns, where residuals are obtained from a daily four-factor model. The control variables include the following. *Beta* is the CAPM beta obtained from estimating a standard daily CAPM data using a six-month rolling window. *Size* is the natural log of end-of-month market capitalization (in $Billions). *B/M* is the natural log of the book-to-market ratio for each stock in each month. *Momentum* is the cumulative return from month *t-12* to *t-2*. *Illiquidity* is the monthly average of the ratio of the absolute value of the daily return scaled by dollar volume (in $Millions). In each month, we sort stocks into terciles based on *MaxRet*. Column 1 reports the results for the bottom tercile. Columns 2 and 3 present the results for middle and top terciles. In parenthesis, we report t-statistics that are obtained from adjusted standard errors that account for three lags.

* denote statistical significance at the 0.10 level.

** denote statistical significance at the 0.05 level.

*** denote statistical significance at the 0.01 level.

In the low *MaxRet* tercile, we find that *Ln(Price Range)* produces a negative coefficient that is not reliably different from zero (estimate = -0.0235, *t*-statistic = -0.48). However, in the high *MaxRet* tercile, the coefficient on *Ln(Price Range)* is -0.4059 (*t*-statistic = -3.20). The difference between these coefficients is statistically significant at the 0.01 level (z-statistic = 2.81) suggesting that consistent with the results in the previous two tables, the return premium associated with range-based volatility is driven by stocks with high maximum daily returns.

As with all of our analysis in Tables 3 through 9, we provide *t-*statistics from robust standard errors [32] that account for three lags. We note that in unreported tests, the conclusions that we draw are similar whether we include zero lags or (up to) six lags. We also note that including *IdioVolt* as an additional control variable in Tables 7 through 9 but does not meaningfully alter our results. Finally, we replicate our analysis by sorting stocks into quintiles based on *E[IdioSkew]* and/or *MaxRet* instead of terciles, and find qualitatively similar results to those reported in this paper. Our analysis shows that range-based volatility is associated with significantly lower returns, is not driven by stocks with higher levels of risk, and is strongest in stocks with lottery-like characteristics.

## Conclusion

Previous research has found conflicting results regarding the cross-sectional association between volatility and future returns. Prior studies [7], [8] have found that idiosyncratic volatility is negatively associated with expected returns in both U.S. and international markets. On the other hand, there is also research that shows that volatility is time-varying and that, when forecasting volatility using exponential GARCH models, the lead-lag, cross-sectional relation between volatility and stock returns becomes positive [10]. These studies indicate that the direction of the relation between risk and return at the firm level depends on how volatility is measured.

In this study, we contribute to the debate by examining a range-based measure of volatility detailed in Alizadeh, Brandt, and Diebold [11]. They show that the natural log of the price range is theoretically, numerically, and empirically superior to other measures of volatility in its efficiency. Compared to other measures of volatility, range-based volatility is also distributed more normally than other measures of volatility and is robust to microstructure issues that are often problematic in volatility estimation.

We conduct a series of traditional asset pricing tests to examine the relation between next-month returns and the natural log of the difference between highest price and the lowest price during a particular month. After sorting stocks into five value-weighted portfolios based on range-based volatility, we find that next-month average returns are monotonically decreasing. The return difference between extreme portfolios is statistically and economically significant. We also estimate portfolio alphas from CAPM and other multifactor models [9], [12] and find that alphas are generally decreasing across increasing range-based volatility portfolios. Next, we examine this cross-sectional, negative return premium for various subsamples based on other measures of risk, specifically, idiosyncratic volatility and beta. While we find that the negative return premium associated with range-based volatility holds in each of the idiosyncratic volatility subsamples, we find strong evidence that the premium is strongest in stocks with the highest idiosyncratic volatility. Finally, we attempt to further identify the explanation for the peculiar negative return premium found in range-based volatility by sorting stocks by lottery characteristics [19]. We test whether the return premium associated with range-based volatility is driven by stocks that are most likely to resemble lotteries. Using the various lottery characteristics from the prior literature, [21], [25], [26] we find that the range-based volatility return premium is driven by stocks that most resemble lotteries.
